# Inhibition of KIF20A by transcription factor IRF6 affects the progression of renal clear cell carcinoma

**DOI:** 10.1186/s12935-021-01879-y

**Published:** 2021-05-03

**Authors:** Xinwei Ma, Xiaoqi Wang, Qian Dong, Hongquan Pang, Jianming Xu, Junkang Shen, Jianbing Zhu

**Affiliations:** 1 Department of Radiology, The Second Affiliated Hospital of Soochow University, No.1055 Sanxiang Road, Suzhou, 215004 Jiangsu China; 2 Department of Radiology, Suzhou Science and Technology Town Hospital, The Affiliated Suzhou Science and Technology Town Hospital of Nanjing Medical University, No.1 Lijiang Road, high Tech Zone, Suzhou, 215153 Jiangsu China; 3 Department of Medical Oncology, The First Affiliated Hospital of Anhui University of Science and Technology, Huainan, 232001 Anhui China

**Keywords:** Renal clear cell carcinoma, IRF6, KIF20A, Prognosis, Early diagnostic target

## Abstract

**Background:**

Renal clear cell carcinoma (ccRCC) is one of the most common malignant tumors, whose incidence is increasing year by year. IRF6 plays an important role in the occurrence of tumors, although there is yet no report on its expression in ccRCC.

**Methods:**

The expression of IRF6 and KIF20A in ccRCC was predicted by GEPIA and HAP databases. In addition, GEPIA database predicted the relationship between IRF6 and KIF20A expressions and the pathological staging, overall survival, and disease-free survival of ccRCC. The possible binding sites of IRF6 and KIF20A promoters were predicted by JASPAR database and verified by luciferase and ChIP assays. The specific effects of IRF6 on ccRCC cell proliferation, invasion and apoptosis were subsequently examined at both cellular level and animal level.

**Results:**

The database predicted down-regulated IRF6 expression in renal carcinoma tissues and its correlation with poor prognosis. IRF6 overexpression inhibited cRCC cell proliferation, invasion and migration. In addition, up-regulated KIF20A expression in renal carcinoma tissues and its association with prognosis were also predicted. Interference with KIF20A inhibited the proliferation, invasion, and migration of ccRCC cells. Finally, we confirmed that KIF20A is a functional target of IRF6 and can partially reverse the effects of IRF6 on the proliferation, invasion and migration of ccRCC cells. Conclusion: Inhibition of KIF20A by transcription factor IRF6 affects cell proliferation, invasion and migration in renal clear cell carcinoma.

**Supplementary Information:**

The online version contains supplementary material available at 10.1186/s12935-021-01879-y.

## Introduction

Renal cell carcinoma, also known as kidney cancer, ranks among the top ten cancers in the world and accounts for 2% of total cancer incidence worldwide with the number increasing every year [1]. Clear cell carcinoma of kidney (ccRCC) originates from proximal renal tubular cells and is the most common type of renal carcinoma, accounting for 70–80% of renal carcinoma [2]. Early stage of ccRCC hardly presents any symptoms [3]. Therefore, research nowadays focuses more on identifying key molecules that may help with the diagnosis and contribute to the treatment of renal clear cell carcinoma.

Interferon Regulatory Factor 6 (IRF6) is a member of the interferon regulatory factor family. The expression product of IRF6 is transcription factor, which can regulate cell proliferation, cycle and differentiation [4–6]. IRF6 plays an important role in tumor genesis and inhibition. A study reported that IRF6 expression is down-regulated in gastric cancer and is associated with poor prognosis, which may be caused by overexpression of ZEB1 and DNA methylation of IRF6 promoter [7]. Decreased expression of IRF6 is also found in highly metastatic nasopharyngeal carcinoma cells, while its overexpression proves to inhibit tumor cell proliferation and growth and enhances their chemotherapeutic sensitivity [8]. However, the expression of IRF6 in ccRCC has not been reported.

The binding sites of IRF6 and KIF20A were predicted by JASPAR2020 database. Through TCGA database mining, Wei et al. found that KIF20A is up-regulated in ccRCC tissues and is of significant correlation with overall survival rate and relapse-free survival rate, and that it is a hub gene associated with metastasis [9]. Six hub genes (CCNB2, CDC20, CEP55, KIF20A, TOP2A and UBE2C) of ccRCC, identified through the co-expression network of GSE40435 and GSE53757 database and PPI, are closely correlated with pathological stage and poor prognosis [10]. According to GSE53757 database, KIF20A is likely to be highly expressed in ccRCC and significantly correlated with prognosis. PCR assay was used to detect the expression profile of 44 patients with ccRCC, through which significant upregulation of KIF20A expression was verified [11]. However, none of these studies functionally described the role of KIF20A overexpression and knockout in ccRCC.

Based on previous research, we investigated the role and mechanism of IRF6 in the malignant progression of ccRCC through database analysis and in vitro and in vivo experimental verification.

## Materials and methods

### Database selection and analysis

Databases used in this study are as follows:

Gene Expression Profiling Interactive Analysis (GEPIA: http://gepia.cancer-pku.cn) for validation of cancer specific expression and prognosis of the IRF6 genes and KIF20A genes.

The Human Protein Atlas (HPA: http://www.proteinatlas.org/) for examination of the expression of IRF6 and KIF20A in renal carcinoma tissues.

JASPAR database (http://www.jaspar.genereg.net)) for prediction of the possible binding sites of the IRF6 and KIF20A promoters.

In addition, in light of the expression of Hub genes and its relationship with pathological stage of tumor in GEPIA, one-way ANOVA statistical analysis was used. The overall survival (OS) and disease free survival analyses of the hub genes were performed using the Mantel-Cox test survival method in GEPIA.

### Animals

6 healthy female BALB/C nude mice aged 6–8 weeks were selected from Animal Experimental Center. All animal experiments in the present study were approved by the Ethics Committee of the Second Affiliated Hospital of Soochow University. All methods were performed in accordance with the United States Public Health Services Guide for the Care and Use of Laboratory Animals, and all efforts were made to minimize the suffering and number of animals used in the present study. The nude mice were raised in SPF conditions. 786-O cells at logarithmic growth stage were inoculated subcutaneously at the right back near the upper limb of the nude mice by 6 × 10^7^ unit/ml. Mice were treated for 4 weeks for modeling. After successful modeling, the mice were sacrificed for cervical dislocation after anesthesia (0.3% pentobarbital sodium, 0.2 ml/10 g, intraperitoneal injection), and subsequent experiments were conducted.

### Cell culture

Renal tubular epithelial cell HK2 cell and renal clear cell carcinoma cell line OS-RC-2, 769-P, CaKi-1, UM-RC-2 and 786-O were purchased from the Cell Resource Center of Shanghai Institutes for Biological Sciences (Chinese Academy of Sciences, Shanghai, China). Cells were cultured in RPMI1640 medium containing 10% fetal bovine serum (FBS), and 100 μg/ml streptomycin at 37 °C with 5% CO_2_ in a humid incubator.

### RT-qPCR

Total RNA was extracted from the cells using TRIzol reagent (Invitrogen, Waltham, Massachusetts). The PrimerScript Real-time reagent kit (TaKaRa, Kusatsu, Shiga, Japan) was used to perform total RNA reverse transcription. SYBR Premix Ex TaqTM II (TaKaRa, Japan) was then used for the quantitation analysis of the expression of IRF6 and KIF20A. We designed the stem-loop with IRF6 and KIF20A sequence (which was used for the primer of reverse transcription) and used it to complete reverse transcription. The primer sequences for primer source are as follows: IRF6: Forward: 5′ CAAAACTGAACCCCTGGAGATGGA 3′ Reverse: 5′- CCACGGTACTGAAAC TTGATGTCC-3′; KIF20A: Forward: 5′-TGCTGTCCGATGACGATGTC-3′ Reverse: 5′-AGGTTCTTGCGTACCACAGAC-3′; GAPDH: Forward: 5′-AGGTTCTTGCGTACCACAGAC-3′ Reverse: 5′-GCCATCACGCCACAGTTTC-3′.

### Western blot

Lysis buffer (Sigma, USA) was added to the cells to isolate total protein. The protein concentration was determined using a bicinchoninic acid assay protein assay kit. Proteins (25 μg/lane) were separated by SDS-10% polyacrylamide gel (Bio-Rad, Hercules, CA) and transferred to polyvinylidene fluoride membrane (Thermo Fisher Scientific, Waltham, MA). The membrane was blocked with 5% milk in Tris-buffered saline/Tween-20 for 1 h at room temperature, and then probed overnight at 4 °C with the following primary antibodies: anti-IRF6 (1:1000; ab123880; Abcam, Cambridge, MA), anti- KIF20A (1:1000; ab7091; Abcam, Cambridge, MA) (Santa Cruz Biotechnology, Santa Cruz, CA), and anti-GAPDH (ab75478; Abcam, Cambridge, MA). After washing, the blots were incubated with HRP-conjugated secondary antibody for 2 h at room temperature. Proteins were detected with the enhanced chemiluminescence detection system (Thermo Fisher Scientific, Waltham, MA). The protein bands were visualized using the ChemiDoc XRS System (Bio-Rad), and the blots were analyzed by Image J software.

### Cell transfection

Overexpression (Oe)-IRF6, Oe-KIF20A, shRNA-IRF6, shRNA-KIF20A and the negative control (NC) were synthesized by GenePharma Co. (Shanghai, China). All cell transfections were conducted using Lipofectamine 3000 reagent (Invitrogen, Carlsbad, CA, USA) following the manufacturer’s protocol.

### CCK-8

786-O cells (1 × 10^5^) were inoculated into 96-well plates. After the cells were treated accordingly, 10 µl CCK-8 solution (Beyotime Institute of Biotechnology) was added to each well at 24, 48 and 72 h time intervals post-transfection. And the plates were then incubated for 48 h at 37 °C. Optical density (OD) values at 450 nm were determined using an ELx808 absorbance microplate reader (BioTek Instruments, Inc., Winooski, VT, USA).

### Colony formation assays

786-O cells (1 × 10^6^) were inoculated into 6-well plates and transfected for 48 h. Then, the transfected cells were prepared into cell suspension and cultured in a 6-well plate with 1 × 10^3^ cell per well for 14 days at 37 °C. After this, the cells were stained with 10% Giemsa (Merck, Germany) for 30 min. Colonies containing ≥ 50 cells were counted under a microscope (Olympus, Japan). Each experiment was repeated three times.

### TUNEL staining

For TUNEL staining, 786-O cells (1 × 10^6^) were inoculated into 6-well plates and transfected for 48 h. Cells were fixed in freshly prepared 4% methanol-free formaldehyde solution in PBS for 20 min at room temperature and permeabilized with 0.2% Triton X-100 for 5 min. 786-O cells were labeled with fluorescein TUNEL reagent mixture for 60 min at 37 °C according to the manufacturer’s suggested protocol. After that, the slides were examined by fluorescence microscopy and the number of TUNEL-positive (apoptotic) cells was counted. DAPI was used to stain nucleus.

### Wound healing

786-O cells (1 × 10^6^) were inoculated into 6-well plates and transfected for 48 h. A wound was created by a 100 µL pipette tip on the cell monolayer. Images were taken at 0 h and 24 h to calculate the % of wound healing.

### Transwell

The treated cells were inoculated into the upper chamber of 24-well Transwell chamber at the density of 2*105 cell/well. The upper chamber was laid with Matrigel glue, while the culture medium with a volume of 600 μL containing 10% FBS was added to the lower chamber. After 24 h of incubation, the Transwell chamber was carefully taken out, and the cells were fixed for 20 min with 4% polyformaldehyde solution. The chamber was then washed with PBS solution, the cells on the surface of the chamber were wiped off with cotton swabs. After staining with crystal violet, observation of the cells was done with a microscope. Six visual fields were selected to photograph and count the cells of each group. The experiment was repeated independently for three times.

### Luciferase assay

Overexpression IRF6 plasmids (or its empty vector control) were co-transfected with wild-type (or mutant) KIF20A -Luc plasmids in cells, allowing for expression for 48 h. The cells were washed with PBS and lysed subsequently. The luciferase activity was measured using a plate reader (BD bioscience) and normalized to the transfection efficiency by using a Renilla luciferase activity kit (pRL-TK). All procedures followed the manufacturers’ instructions. All plasmids were constructed by Life Technologies Corporation (Carlsbad, CA).

### Chromatin immunoprecipitation assay (ChIP).

Chromatin immunoprecipitation (ChIP) was carried out with a Magna Chromatin Immunoprecipitation kit (Millipore, Darmstadt, Germany). Immunoprecipitation was performed with anti-IRF6 antibody. The purified DNA fragment was subjected to PCR analysis using Hot-Start Taq DNA polymerase (TaKaRa, Dalian, China; 32 cycles). PCR products were analyzed using gel electrophoresis. ChIP data are shown as the percentage of the input normalized to control purifications.

### Immunohistochemistry (IHC)

Xenograft tumors were collected, sliced and embedded with paraffin. 4 μm-thick sections were deparaffinized, rehydrated and then immersed with 3% hydrogen peroxide for 10 min to quench endogenous peroxidase and labeled with antibodies at 4 °C overnight. The slides were stained with the secondary streptavidin–horseradish peroxidase-conjugated antibody (Santa Cruz Biotech, Santa Cruz, CA) for 1 h, followed by counterstain with hematoxylin for 30 s.

### Statistical analysis

Statistical calculations were performed using SPSS 13.0 software (SPSS, Inc., Chicago, IL, USA). The data are presented as the mean ± standard deviation from at least three independent experiments. Statistical analysis was performed by one-way ANOVA. P < 0.05 is considered to indicate a statistically significant difference.

## Results

### Expression of IRF6 in renal carcinoma tissues and its relationship with prognosis

**Fig. 1:**
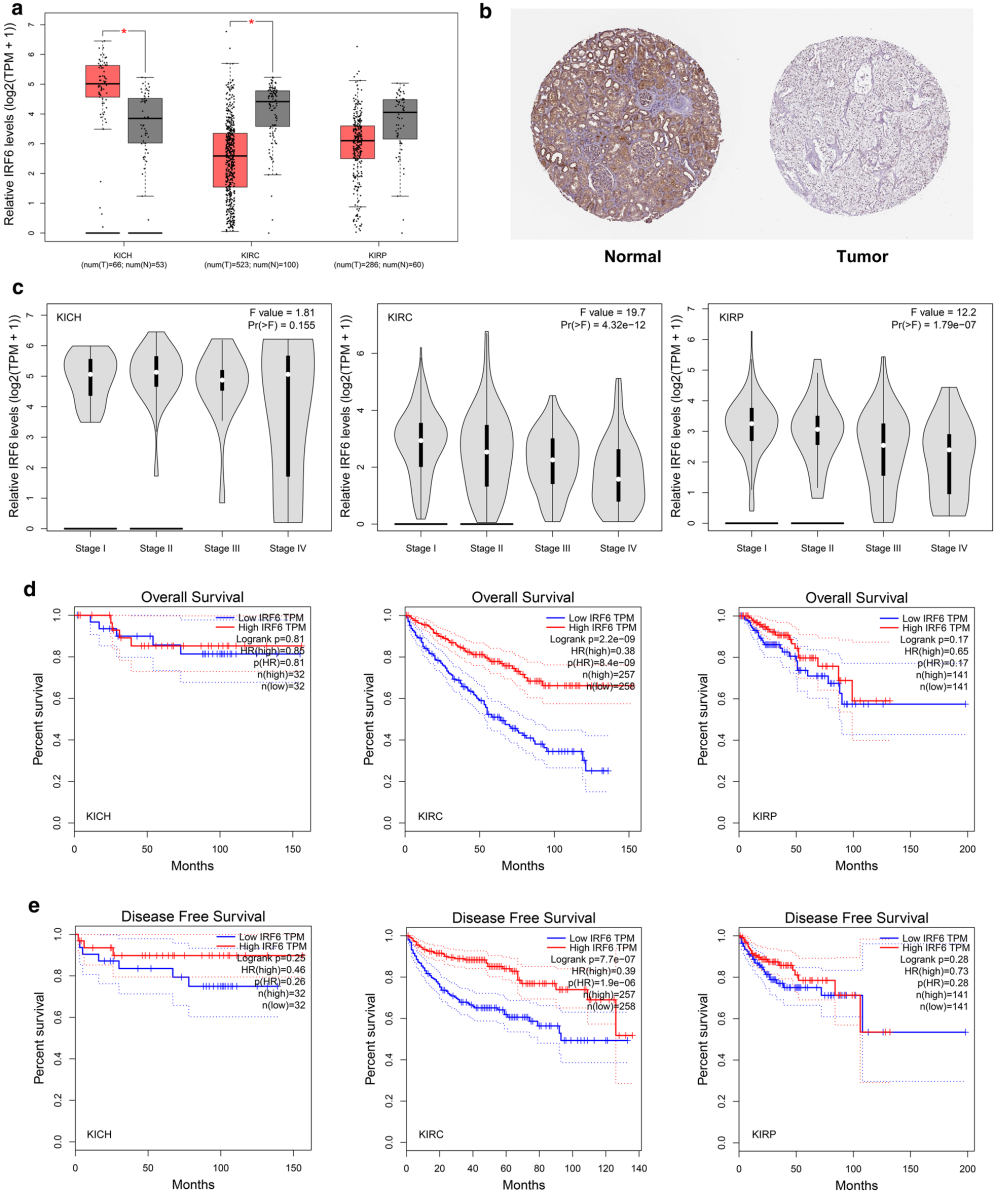
Expression of IRF6 in renal carcinoma tissues and its relationship with prognosis. **a** Comparison of the gene expression levels of IRF6 between normal kidney and ccRCC samples based on TCGA data in GEPIA. **b** IHC staining of IRF6 expression in ccRCC cancer tissues and in normal ccRCC tissue. **c** Validation of the correlation between the expression levels of IRF6 and the pathologic stage of ccRCC. **d** Overall survival analysis of IRF6 in ccRCC (based on TCGA data in GEPIA). **e** Disease free survival analysis of IRF6 in ccRCC (based on TCGA data in GEPIA). Red line represents the samples with high gene expression and blue line for the samples with low gene expression. HR: hazard ratio

Through GEPIA database, we predicted the expression of IRF6 in the tissues of patients with kidney chromophobe (KICH), kidney renal clear cell carcinoma (KIRC), and kidney renal papillary cell carcinoma (KIRP). The expression of IRF6 was significantly decreased in KIRC patients (Fig. 1a). HPA database showed that the IHC results of IRF6 in renal carcinoma were negative (Fig. 1b). In addition, our investigation on the basis of GEPIA data demonstrated that IRF6 expression was correlated strongly with the pathological stage of renal clear cell carcinoma (Fig. 1c). In view of the fact that tumor progression often affects tumor prognosis, we also investigated the roles of IRF6 in ccRCC prognosis including overall survival time and disease-free survival time. We found a shorter overall survival time (Fig. 1d) and disease-free survival time in patients with lower expression levels of IRF6 (Fig. 1e). These results indicated that the expression of IRF6 was low in ccRCC, and was closely related to the prognosis of ccRCC.

### Overexpression of IRF6 inhibits the proliferation, invasion and migration of ccRCC cells

**Fig. 2:**
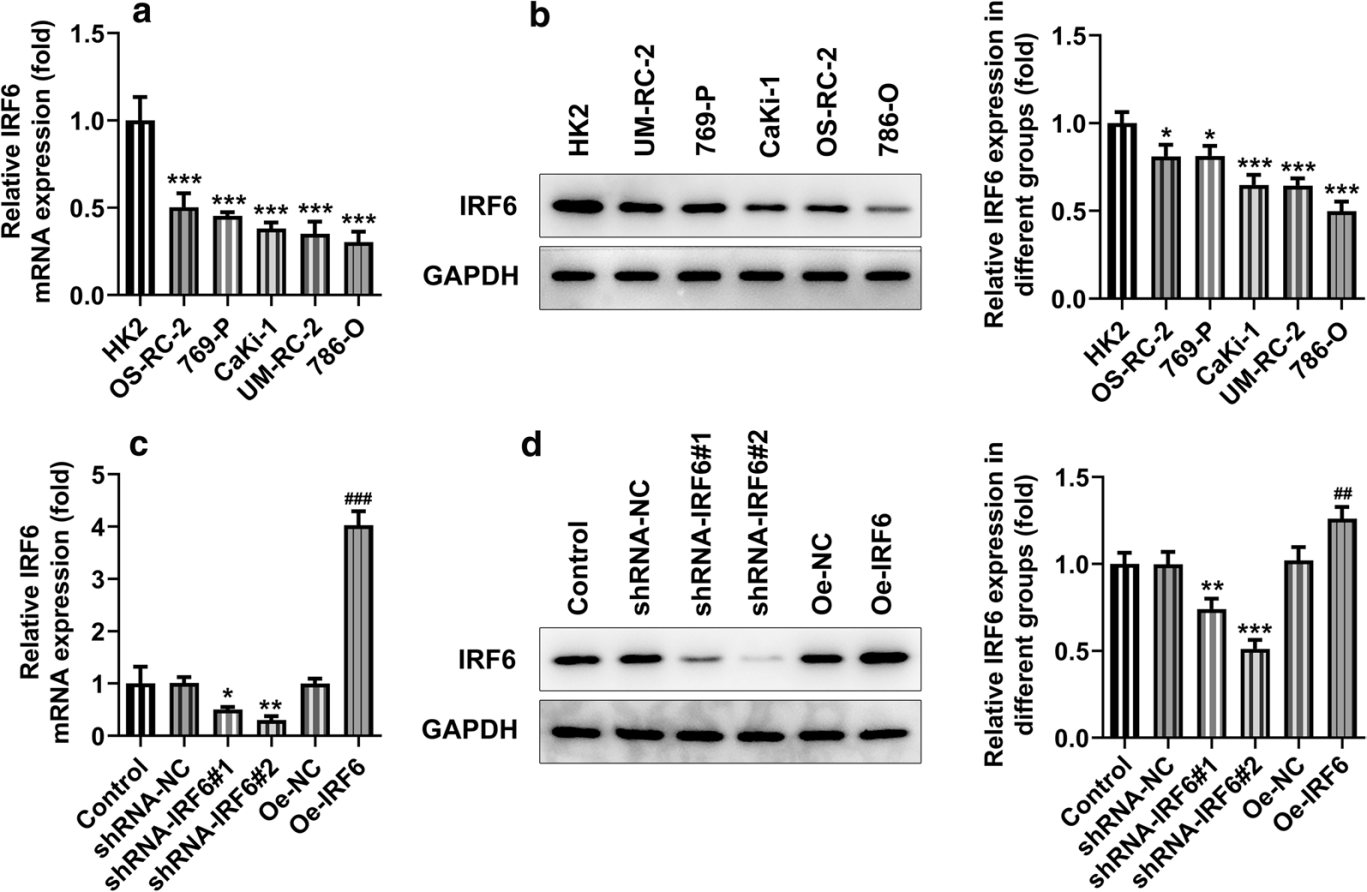
Expression of IRF6 in ccRCC cancer cell lines. **a** RT-qPCR detected the expression of IRF6 in different ccRCC cancer cell lines. **b** Western blot detected the expression of IRF6 in different ccRCC cancer cell lines. *P < 0.05, ***P < 0.001 vs HK2. **c** The expression of IRF6 in cells was detected by RT-qPCR after cell transfection. **d** The expression of IRF6 in cells was detected by western blot after cell transfection. *P < 0.05, **P < 0.01, ***P < 0.001 vs shRNA-NC. ^##^P < 0.01, ^###^P < 0.001 vs Oe-NC

**Fig. 3:**
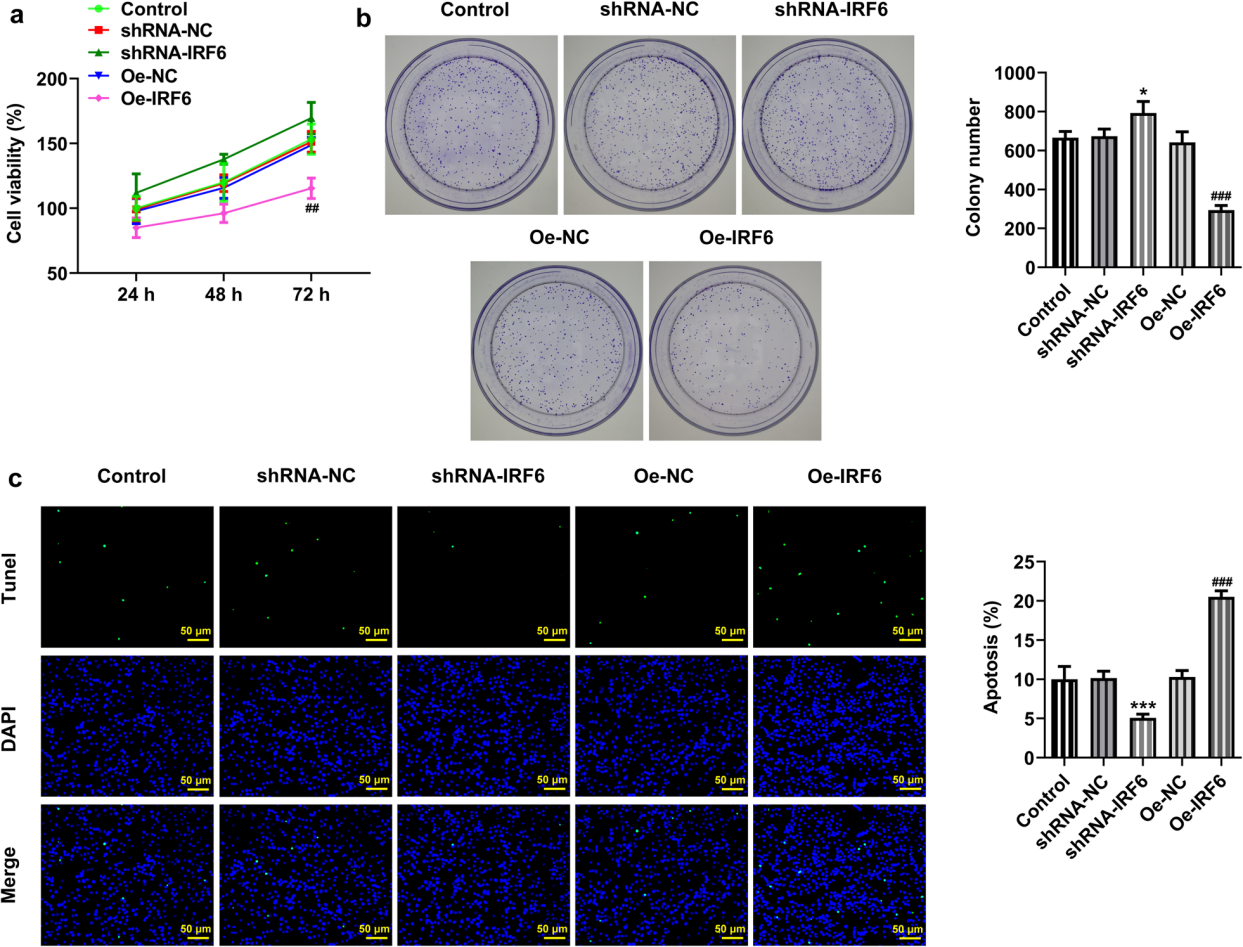
Overexpression of IRF6 inhibits the proliferation of ccRCC cells. **a** CCK-8 detected the cell viability. **b** Clone formation assay detected the cell reproductive capacity. **c** TUNEL assay detected the apoptosis of cells. *P < 0.05, ***P < 0.001 vs shRNA-NC. ^###^P < 0.001 vs Oe-NC

**Fig. 4:**
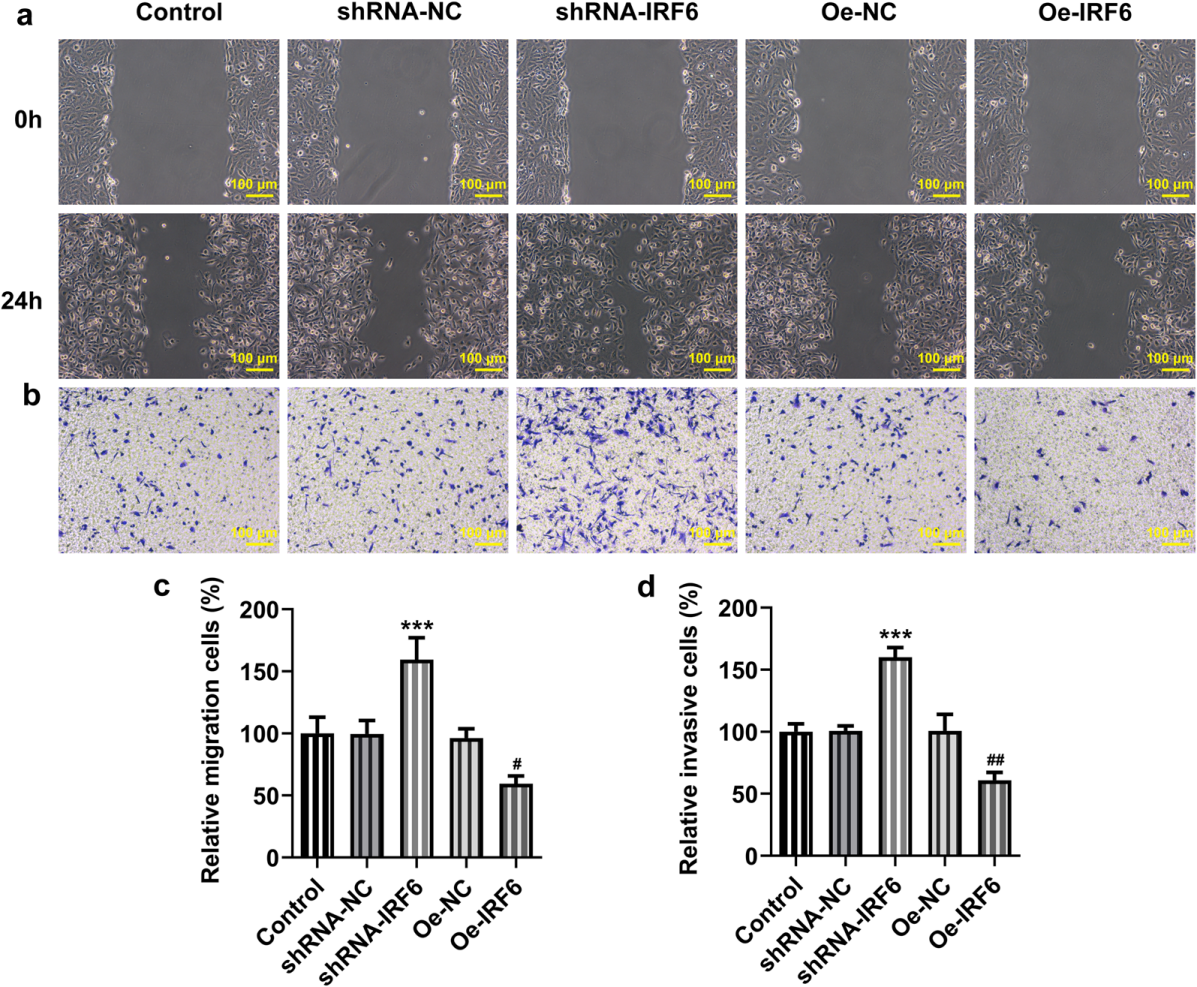
Overexpression of IRF6 inhibits the invasion and migration of ccRCC cells. **a** Wound healing detected the cell migration ability. **b** Transwell detected the cell invasion ability. **c** Statistical chart of cell mobility. **d** Statistical chart of cell invasion. ***P < 0.001 vs shRNA-NC. ^#^P < 0.05, ^##^P < 0.01 vs Oe-NC

We then validated our prediction at the cellular level. The results showed that the expression of IRF6 was significantly decreased in ccRCC cell lines (Fig. 2a, b). We selected 786-O cells for subsequent experiments. Cell transfection assay was performed to interfere with IRF6 expression and overexpress IRF6. As shown in Fig. 2c, d, shRNA-IRF6 #2 had a better interference effect and was thus selected for the subsequent experiments. The expression of IRF6 in cells increased significantly after overexpression of IRF6, indicating successful construction of the overexpression plasmid. We divided the cells into control, shRNA-NC, shRNA-IRF6, Oe-NC and Oe-IRF6 groups. Through detection of cell proliferation, we found that the proliferation ability of 786-O cells was significantly increased after the interference with IRF6 expression but decreased after the overexpression of IRF6 (Fig. 3a, b). Cell apoptosis was detected by TUNEL assay, and the result showed that the apoptosis of 786-O cells was decreased in the shRNA-IRF6 group compared with the shRNA-NC group. Compared with the Oe-NC group, the apoptosis rate of the Oe-IRF6 group was increased (Fig. 3c). The result of wound healing experiment showed that after the interference with IRF6 expression, the cell migration ability was significantly improved, whereas it was dampened after the overexpression of IRF6 (Fig. 4a, c). The result of Transwell showed the same trend with that of the wound healing experiment (Fig. 4b, d). These results indicated that overexpression of IRF6 could inhibit proliferation, invasion and migration of CCRCC cells.

### KIF20A partially reverses the effects of IRF6 on the proliferation, invasion and migration of ccRCC cells

**Fig. 5:**
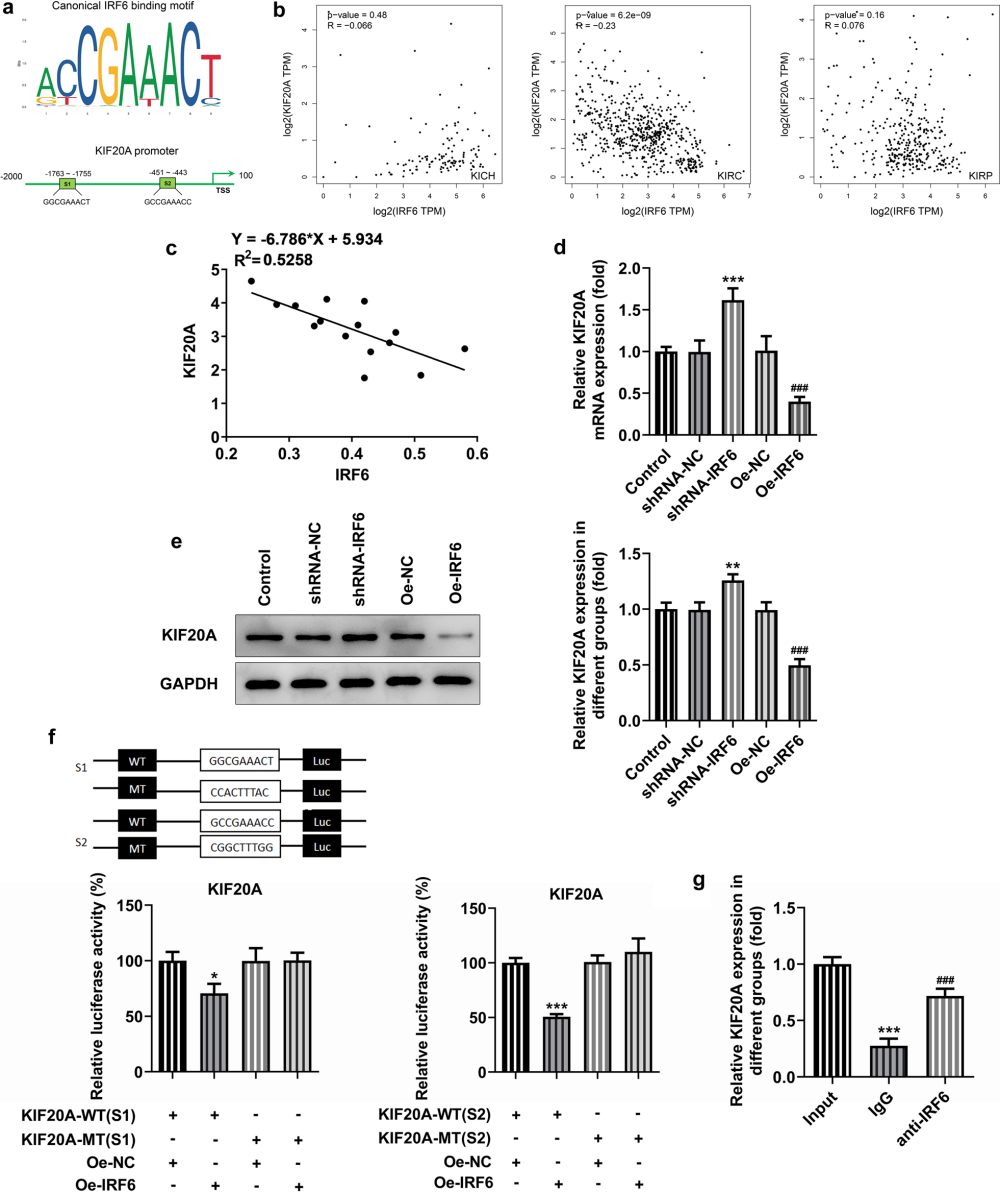
IRF6 directly binds to the KIF20A promoter to regulate KIF20A expression. **a** JASPAR database predicts binding sites between IRF6 and KIF20A. **b** Correlation between IRF6 and KIF20A expression in ccRCC. **c** The correlation between IRF6 and KIF20A (the qPCR expression). **d** RT-qPCR detected the expression of KIF20A. **e** Western blot detected the expression of KIF20A. **P < 0.01, ***P < 0.001 vs shRNA-NC. ^###^P < 0.001 vs Oe-NC. **f** Luciferase was used to detect the transcriptional activity of KIF20A promoter mutant in ccRCC cells. *P < 0.05 vs KIF20A-WT + Oe-IRF6. **g** ChIP verified that IRF6 binds to the KIF20A promoter. ***P < 0.001 vs Input. ^###^P < 0.001 vs IgG

We used JASPAR database to predict the binding sites (S1 and S2) of IRF6 and KIF20A promoters (Fig. 5a). KIF20A plays an important role in cancer prognosis. In our supplemental data, we detected increased expression of KIF20A in renal carcinoma tissues and it was strongly associated with prognosis of renal clear cell carcinoma (Additional file 1: Fig S1). Interference with KIF20A can significantly inhibit proliferation, invasion and migration of renal clear cell carcinoma (Additional file 1: Fig S2–4). GEPIA database showed that the expression levels of IRF6 and KIF20A were closely correlated with each other in KIRC (Fig. 5b). We also studied the high correlation between IRF6 and KIF20A in five ccRCC cell lines (Fig. 5c). The expression of KIF20A in ccRCC cells was significantly increased by interference with IRF6, while the change was reversed after overexpression of IRF6 (Fig. 5d, e). Luciferase assay detected the transcriptional activity of KIF20A promoter mutant in ccRCC cells (Fig. 9f). Finally, ChIP experiment verified the combination of IRF6 and KIF20A promoter (Fig. 9g). The above experimental results indicate that KIF20A is a functional target of IRF6 (Additional file 2).

**Fig. 6:**
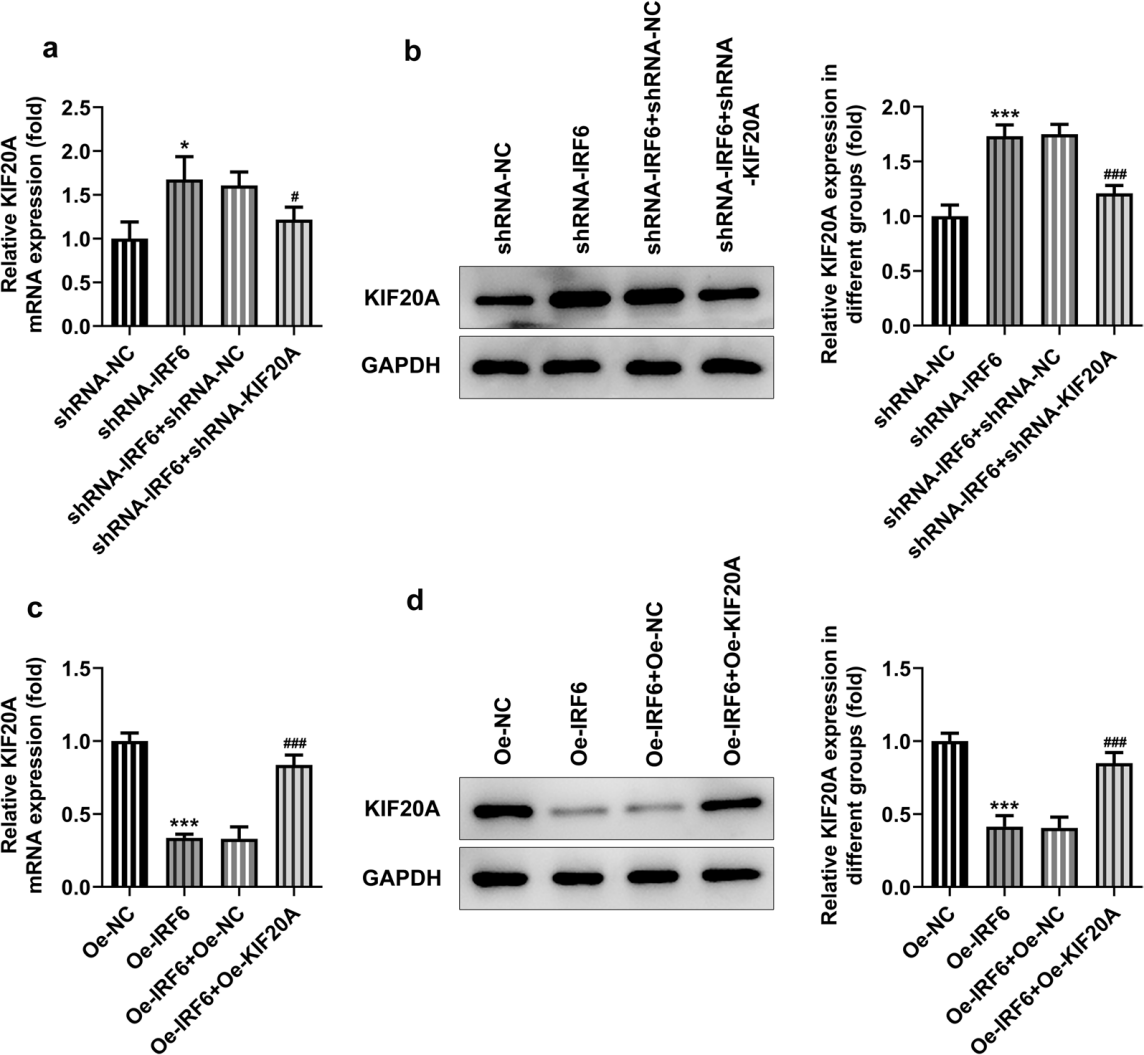
Expression of KIF20A after cell transfection. **a** RT-qPCR detected the expression of KIF20A after inhibition of IRF6 and KIF20A expression. **b** Western blot detected the expression of KIF20A after inhibition of IRF6 and KIF20A expression. *P < 0.05, ***P < 0.001 vs shRNA-NC. ^#^P < 0.05, ^###^P < 0.001 vs shRNA-IRF6 + shRNA-NC. **c** RT-qPCR detected the expression of KIF20A after overexpression of IRF6 and KIF20A. **d** Western blot detected the expression of KIF20A after overexpression of IRF6 and KIF20A. ***P < 0.001 vs Oe-NC. ^###^P < 0.001 vs Oe-IRF6 + Oe-NC

**Fig. 7:**
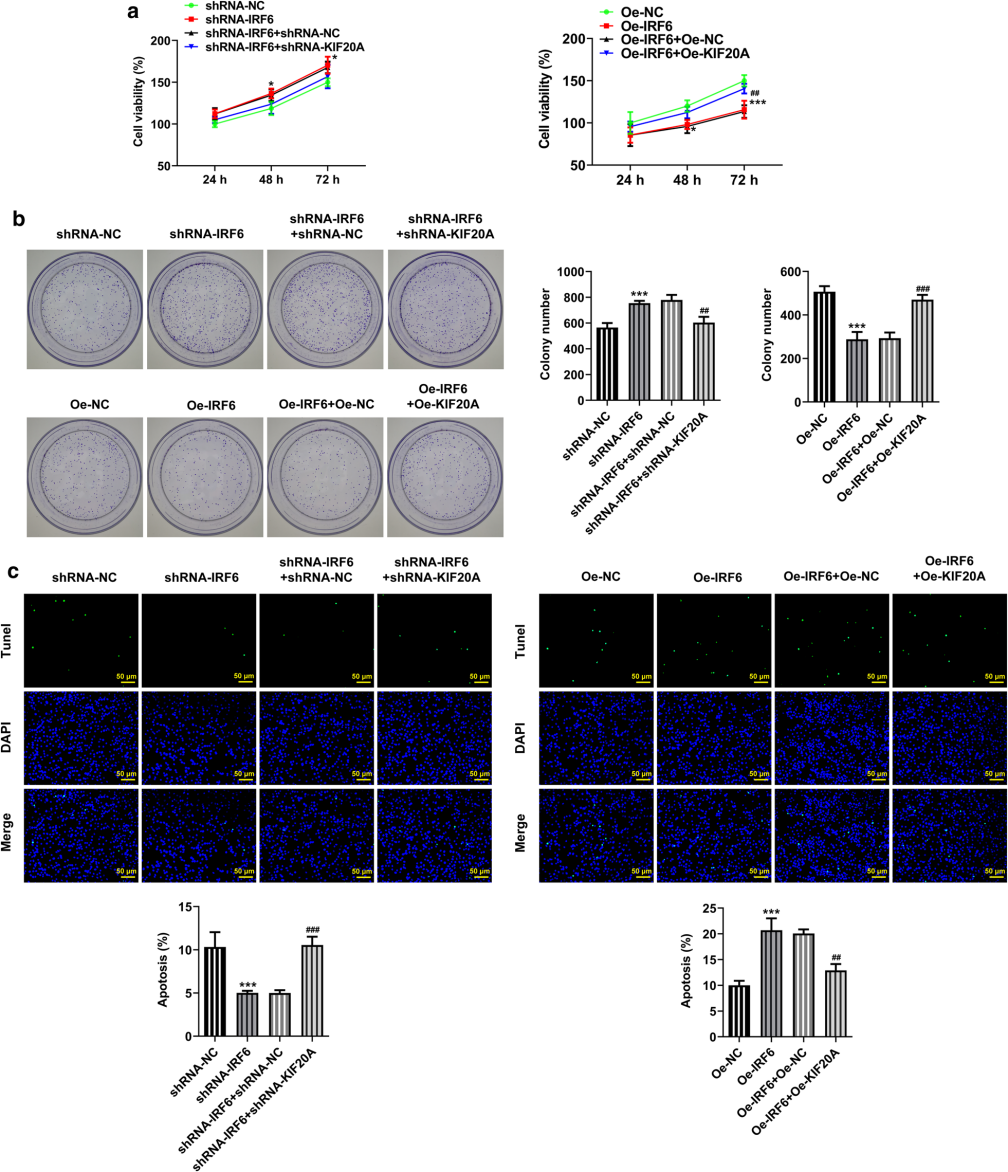
KIF20A partially reverses the effects of IRF6 on the proliferation of ccRCC cells. **a** CCK-8 detected the cell viability. **b** Clone formation assay detected the cell reproductive capacity. c TUNEL assay detected the apoptosis of cells. *P < 0.05, ***P < 0.001 vs Oe-NC. ^##^P < 0.01, ^###^P < 0.001 vs Oe-IRF6 + Oe-NC

**Fig. 8:**
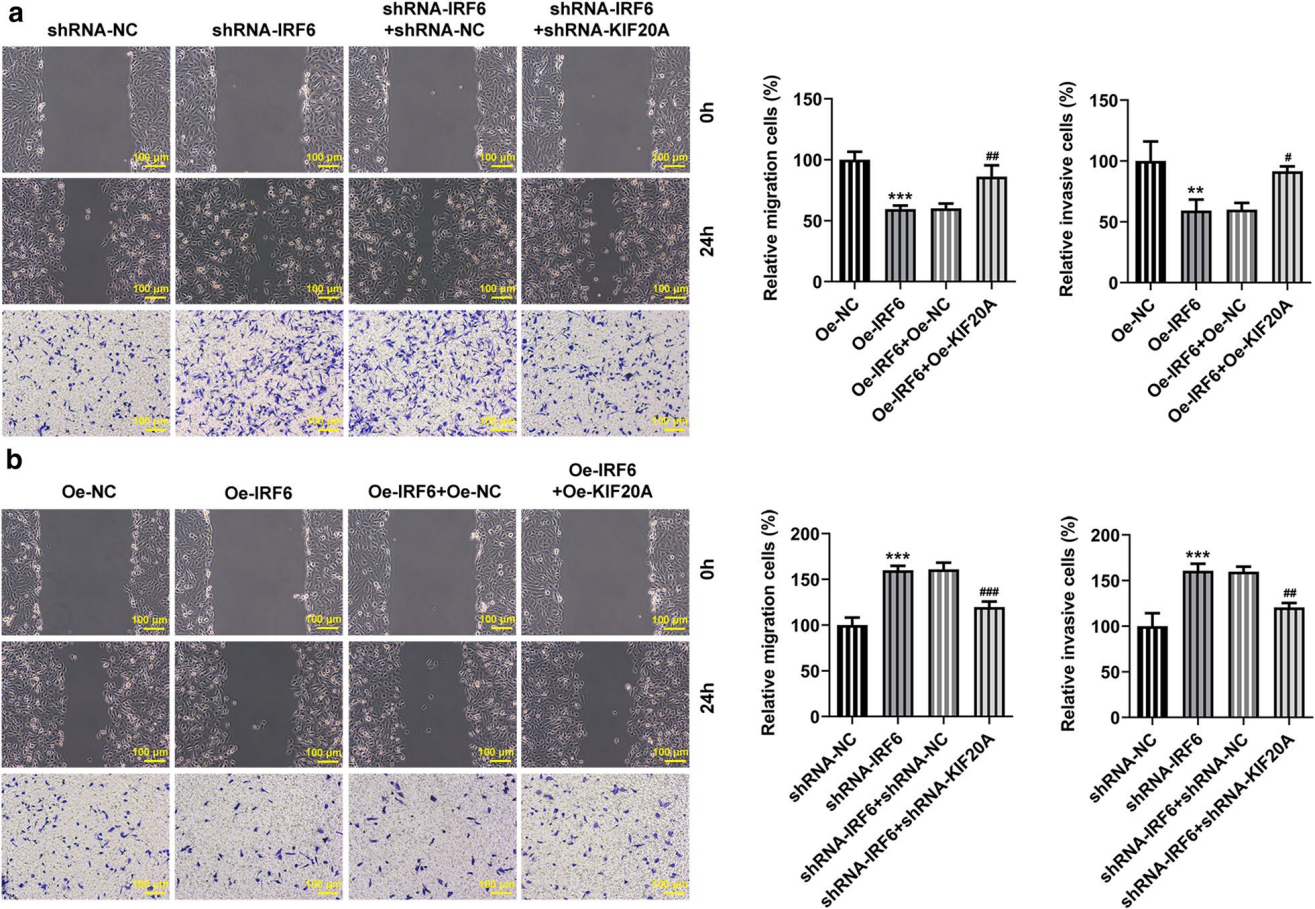
KIF20A partially reverses the effects of IRF6 on the invasion and migration of ccRCC cells. **a** Wound healing detected the cell migration ability. **b** Transwell detected the cell invasion ability. **P < 0.01, ***P < 0.001 vs Oe-NC. ^#^P < 0.05, ^##^P < 0.01, ^###^P < 0.001 vs Oe-IRF6 + Oe-NC

The above experimental results led us to speculate whether IRF6 plays a role in proliferation, invasion and migration of ccRCC by regulating the expression of KIF20A. Cells were divided into the following groups: shRNA-NC, shRNA-IRF6, shRNA-IRF6 + shRNA-NC and shRNA-IRF6 + shRNA-KIF20A; Oe-NC, Oe-IRF6, Oe-IRF6 + Oe-NC and Oe-IRF6 + Oe-KIF20A. We found that the expression of KIF20A increased after IRF6 interference, which was reversed after further inhibition of KIF20A expression (Fig. 6a, b). Conversely, the expression of KIF20A decreased after overexpression of IRF6, which was reversed by KIF20A overexpression in the cells (Fig. 6c, d). Increased cell viability (Fig. 7a), proliferation (Fig. 7b), migration and invasion (Fig. 8a) in addition to decreased cell apoptosis (Fig. 7c) were found as the interference with IRF6 expression up-regulated the expression of KIF20A. Further down-regulation of KIF20A expression in cells inhibited cell proliferation, invasion and migration (Fig. 8b) and promoted cell apoptosis. IRF6 overexpression down-regulated the expression of KIF20A and further attenuated cell viability, proliferation, migration and invasion (Fig. 8b), while promoting cell apoptosis. These trends were all reversed later after upregulation of KIF20A expression in ccRCC cells. The experiment result shows that KIF20A can partially reverse the effects of IRF6 on the proliferation, invasion and migration of ccRCC cells.

### Overexpression of IRF6 inhibits the growth and metastasis of ccRCC in vivo

**Fig. 9:**
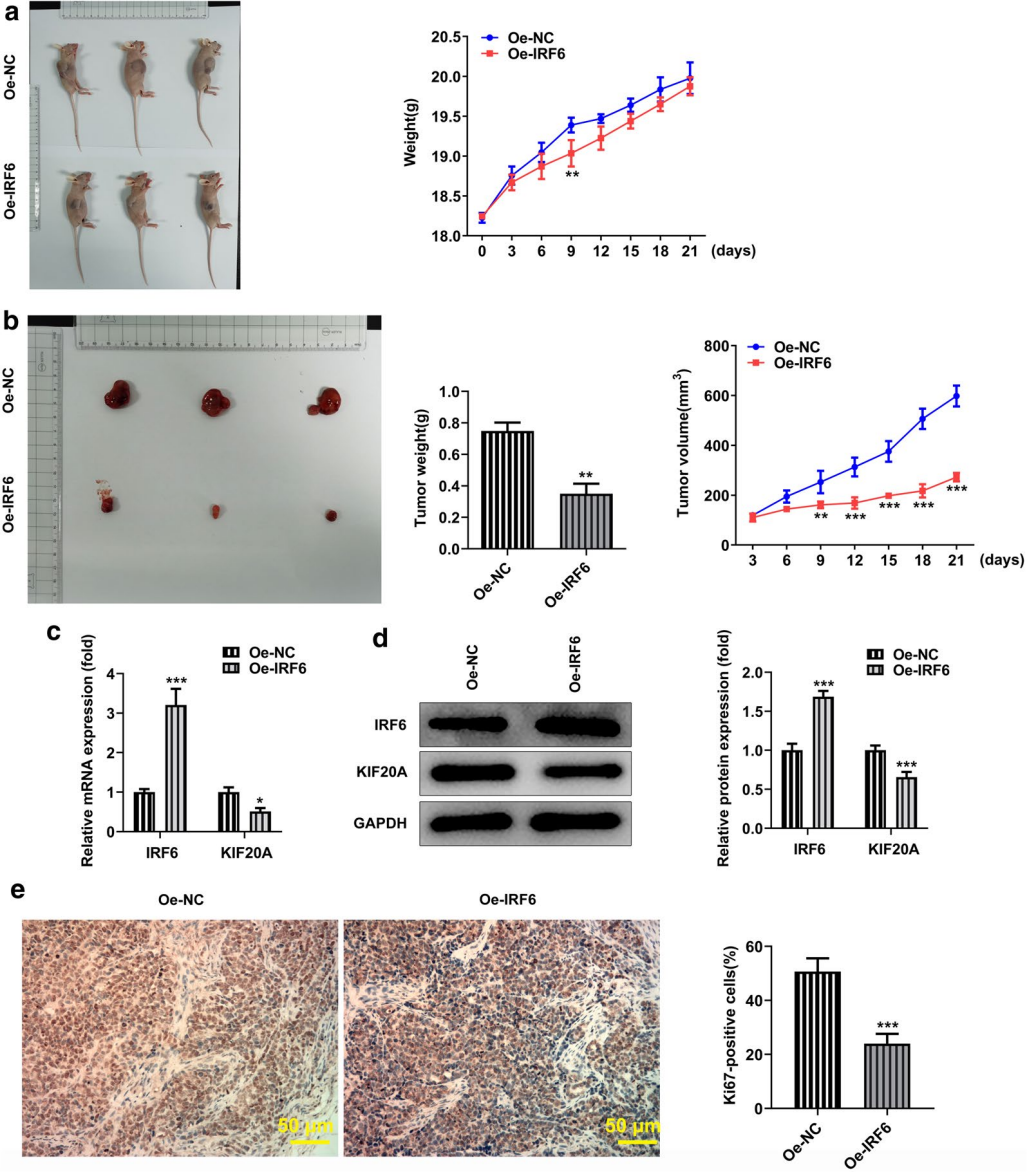
Overexpression of IRF6 inhibits the growth and metastasis of ccRCC in vivo. **a** Mice were weighed and photographed before treatment. **b** The weight and volume of tumor tissue. **c** RT-qPCR detected the expression of IRF6. **d** Western blot detected the expression of IRF6. **e** The expression of ki-67 in tumor tissues was detected by IHC

In order to further verify our experimental results, we carried out experiments on animals. Mice were weighed and photographed before treatment (Fig. 9a). Then the tumors were taken off from the mice, weighed and photographed followed by measurement of the volume, as shown in Fig. 9b. It was found that the tumor weight and volume decreased after the overexpression of IRF6. The expression of IRF6 and KIF20A in tumor tissues was detected by RT-QPCR (Fig. 9c) and western blot (Fig. 9d). After the overexpression of IRF6, the expression of IRF6 in tumor tissues increased, while the expression of KIF20A decreased. In addition, the expression of Ki67 was detected by IHC and was found to be significantly decreased in the Oe-IRF6 group, compared with Oe-NC (Fig. 9e), indicating that the overexpression of IRF6 inhibits the proliferation of tumor cells in tumor-bearing mice.

## Discussion

At present, molecular markers of renal clear cell carcinoma mainly include hypoxia-inducing factors HIF [12] and P53 [13], etc. Unfortunately, these molecular markers have certain limitations and cannot be widely used in clinical practice. Therefore, efforts are being made to find new molecular markers that can contribute to early diagnosis and treatment of ccRCC.

IRF6 regulates craniofacial development and epidermal hyperplasia [5]. In recent years, abnormal expression of IRF6 has been proved to cause a variety of diseases and regulate various physiological and biochemical processes [14]. IRF6 plays an important role in the development of tumors. Down-regulation of IRF6 expression can inhibit the differentiation of primary human keratinocytes in vivo and in vitro and promote the formation of RAS-induced tumor [15]. Martha L et al. found that IRF6 is negatively associated with colorectal cancer risk and survival [16]. However, the expression of IRF6 in ccRCC and its relationship with cancer prognosis have not been reported. In our study, we found significantly down-regulated IRF6 expression in ccRCC tissues through GEPIA and HAP databases. In addition, our basic experiments verified this finding, as IRF6 expression was down-regulated in ccRCC mice as well as ccRCC cell lines.

Study has shown that IRF6 can be used as the downstream of Notch signaling pathway to regulate the proliferation and transformation of breast cancer cells, and can also be used as a potential susceptibility marker of breast cancer [17, 18]. Markedly decreased IRF6 expression is found in cutaneous squamous cell carcinoma and has been shown to promote the invasion and growth of cancer cells [19]. These results indicate that IRF6 can inhibit the proliferation, invasion and migration of tumor cells in a variety of tumors. Our study consistently demonstrated that overexpression of IRF6 can effectively inhibit ccRCC cell proliferation, invasion and migration and promote its apoptosis.

JASPAR predicts that IRF6 can target KIF20A. The relevant experiments in our study verified this prediction as they showed that IRF6 can directly bind to the KIF20A promoter to regulate KIF20A expression. KIF20A, a member of Kinesin6 family, is involved in key cellular functions including intracellular activity of organelles and vesicles, spindle formation and cytokinesis [20]. The expression and activity of KIF20A participate in the regulation of intracellular transport and cell division, playing an important role in the occurrence and development of cancer [21]. KIF20A can affect the prognosis of bladder cancer by promoting the proliferation and metastasis of bladder cancer cells.KIF20A [22]. In soft tissue sarcoma, KIF20A gene knockout can inhibit the proliferation, migration and invasion of soft tissue sarcoma cells, promote cell apoptosis, and inhibit tumor growth [23]. These results indicated that KIF20A had an important effect on the proliferation, invasion and apoptosis of tumor cells. In our study, we found that the expression of KIF20A was significantly increased in ccRCC, and that interfering with KIF20A inhibited cell proliferation, invasion and migration. In addition, KIF20A can partially reverse the effects of IRF6 on the proliferation, invasion and migration of ccRCC cells. In addition, KIF20A is a candidate biomarker for prognosis of non-small cell lung cancer (NSCLC) [24], glioblastoma [25] and liver cancer [26]. In our supplemental data, we also found a significant correlation between KIF20a and ccRCC tumor pathological staging and overall survival.

Through GEPIA, HAP and other databases, we found that the expression of IRF6 and KIF20A in ccRCC is likely to be correlated with the pathological stage and overall survival rate of renal carcinoma patients. These results suggest that IRF6 and KIF20A may serve as prognostic markers for poor survival in ccRCC patients.

In this paper, we have confirmed that IRF6 directly regulates the expression of KIF20A by binding with the KIF20A promoter, and the expression of IRF6 and KIF20A is correlated with the pathological staging and overall survival of patients with ccRCC. Therefore, it is reasonable to speculate that IRF6 targets to regulate KIF20A and thus plays a regulatory role in the proliferation, invasion, migration and apoptosis of ccRCC tumors. In our study, we found that KIF20A partially reversed the effects of IRF6 on the proliferation, invasion, migration and apoptosis of CCRCC cells.

## Conclusion

It is demonstrated in the present study that the transcription factor IRF6 inhibits the expression of KIF20A and thus affects the proliferation, invasion and apoptosis of ccRCC cells.

## Supplementary Information

**Additional file 1.** Role of KIF20A in ccRCC.

**Additional file 2.** Original dates.

## Data Availability

We hereby undertake that all data and materials are available.
