# Supplementary material for: Inhibition of KIF20A by transcription factor IRF6 affects the progression of renal clear cell carcinoma
Source: Cancer Cell Int. 2021 May 3;21:246. doi: 10.1186/s12935-021-01879-y (PMC8091794; doi:10.1186/s12935-021-01879-y)
Supplement: Supplementary file 1 — Additional file 1. Role of KIF20A in ccRCC. [file 12935_2021_1879_MOESM1_ESM.docx]

**Expression of KIF20A in renal carcinoma tissues and its relationship with prognosis**

Through the GEPIA database, we predicted the expression of KIF20A in the tissues of KICH, KIRC and KIRP patients, and the results were shown in Figure S1A. The expression of KIF20A was significantly increased in KIRC patient tissues. HPA database results showed that KIF20A was highly expressed in renal cancer tissues (FigS1B). In addition, based on GEPIA data, we investigated that KIF20A expression was highly correlated with the pathological stage of renal clear cell carcinoma (FigS1C). We also investigated the roles of KIF20A in ccRCC prognosis including overall survival time and disease free survival time. We found a shorter overall survival time (FigS1D) and disease-free survival time in patients with higher expression levels of KIF20A (FigS1E).

**Interference with KIF20A inhibits proliferation, invasion and migration of ccRCC cells**

Our results showed that KIF20A was significantly increased in ccRCC cell lines (FigS2A and B). Cell transfection was used to disrupt KIF20A expression and construct KIF20A overexpressed plasmid. The transfection efficiency was detected by RT-qPCR and western blot. The interference effect of shRNA-KIF20A #2 was better, so shRNA-KIF20A #2 was selected for subsequent experiments. In addition, the expression of KIF20A in cells increased significantly after overexpression of KIF20A, indicating successful construction of the overexpressed plasmid (FigS2C and D). We divided the cells into control, shRNA-NC, shRNA-KIF20A, Oe-NC and Oe-KIF20A. Cell proliferation was detected, and we found that the cell proliferation ability decreased significantly after interfering of KIF20A expression, while the cell proliferation ability increased after the over-expression of KIF20A (FigS3 A and B). Apoptosis was detected by TUNEL assay. Compared with the shRNA-NC, apoptosis was increased in the shRNA-KIf20A group. Compared with the Oe-NC, the apoptosis rate of the Oe-IRF6 group was decreased (FigS3 C). The results of wound healing (FigS4 A and C) and transwell (FigS4 B and D) showed that the cell migration and invasion decreased significantly after interfering of KIF20A, while increased after the over-expression of KIF20A.

**
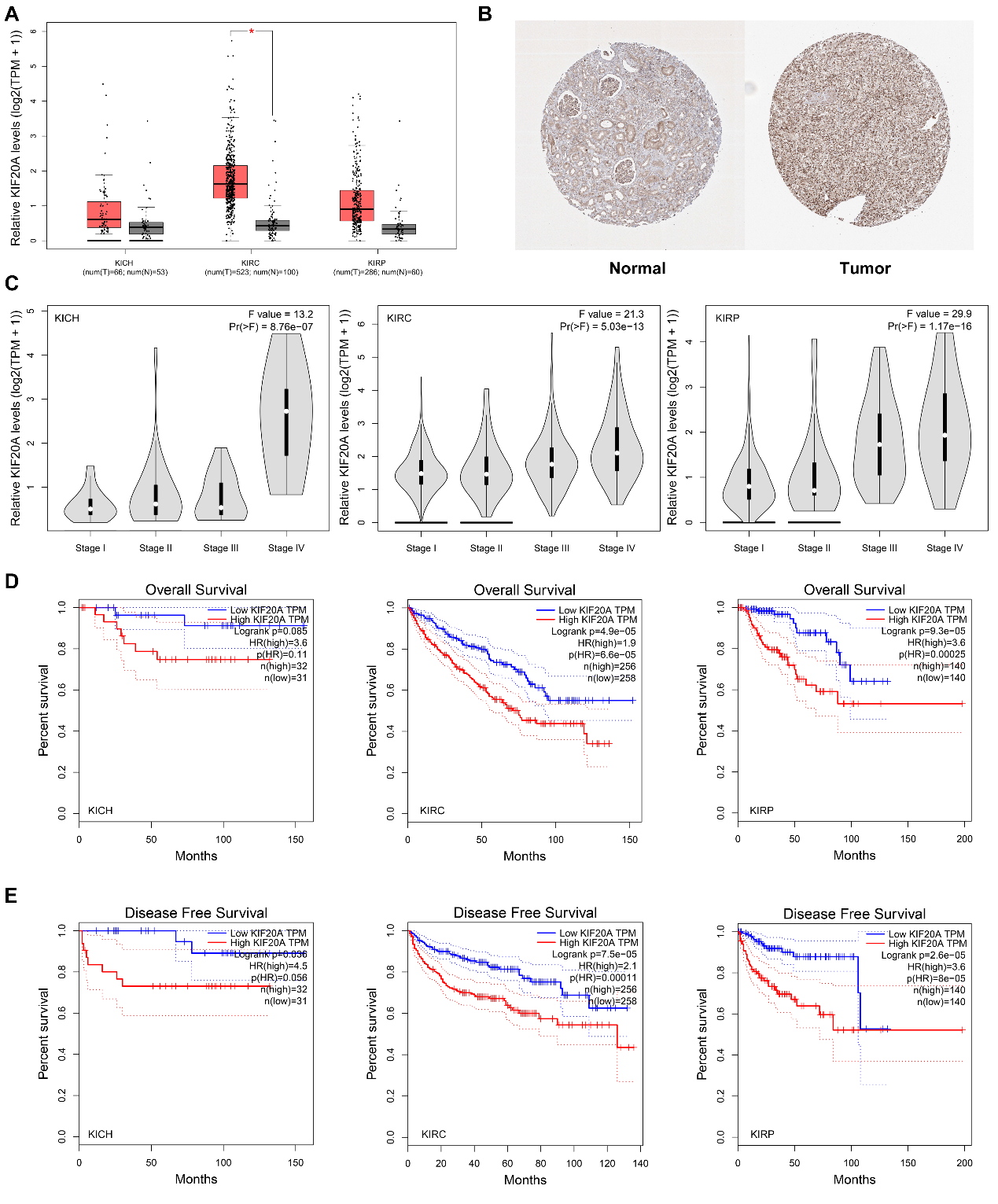
**

**FigS1:** Expression of KIF20A in renal carcinoma tissues and its relationship with prognosis. A. Validation of the gene expression levels of KIF20A between normal kidney and ccRCC samples based on TCGA data in GEPIA. B. IHC staining of KIF20A expression in ccRCC cancer tissues and in normal ccRCC tissue. C. Validation of the correlation between the expression levels of KIF20A and the pathologic stage of ccRCC.  D. Overall survival analysis of KIF20A in ccRCC (based on TCGA data in GEPIA). E. Disease free survival analysis of KIF20A in ccRCC (based on TCGA data in GEPIA). Red line represented the samples with gene highly expressed and blue line was for the samples with gene lowly expressed. HR: hazard ratio.

**
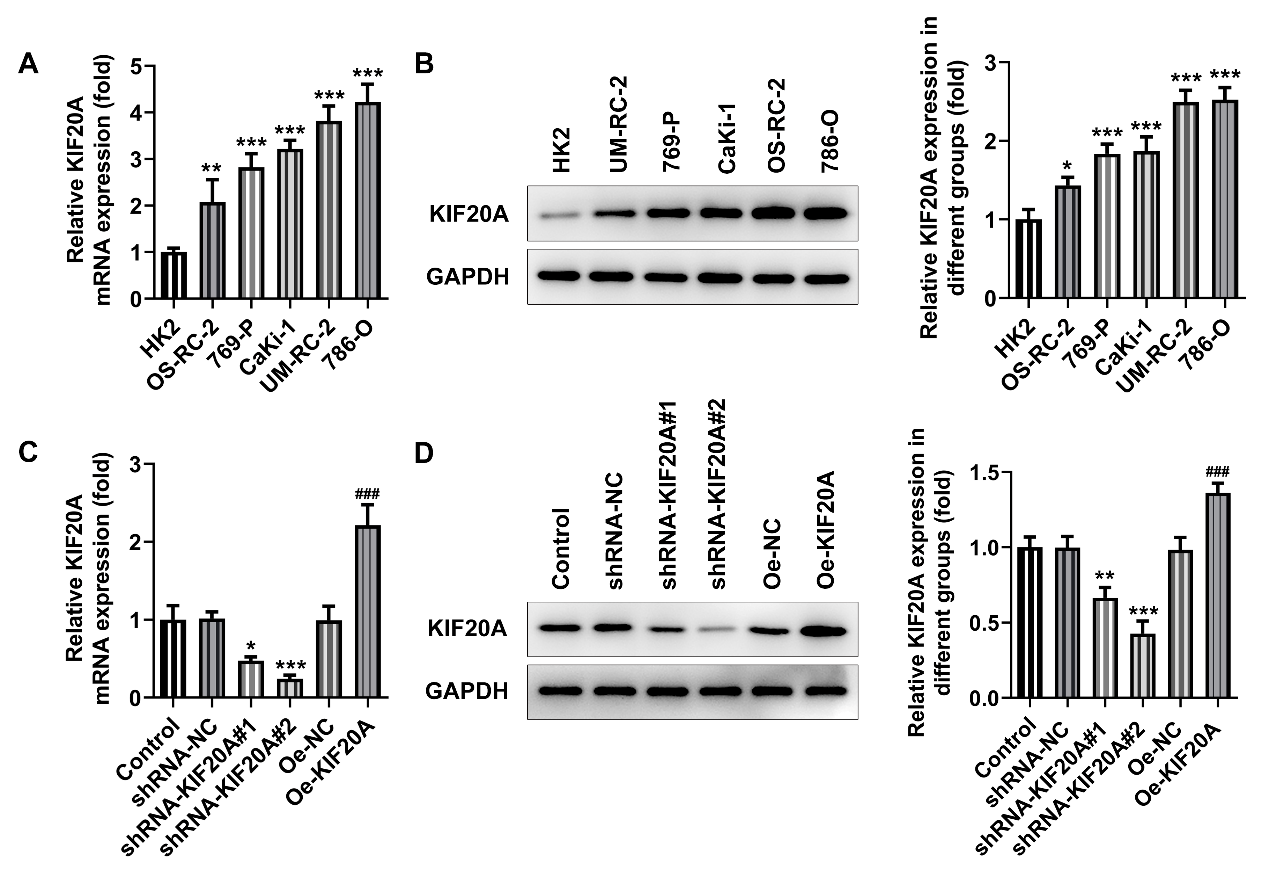
**

**FigS2:** Expression of KIF20A in ccRCC cancer cell lines. A. RT-qPCR detected the expression of KIF20A in different ccRCC cancer cell lines. B. Western blot detected the expression of KIF20A in different ccRCC cancer cell lines. *P<0.05, ***P<0.001 vs HK2. C. The expression of IRF6 in cells was detected by RT-qPCR after cell transfection. D. The expression of KIF20A in cells was detected by western blot after cell transfection. *P<0.05, **P<0.01, ***P<0.001 vs shRNA-NC. ## P<0.01, ### P<0.001 vs Oe-NC.

**
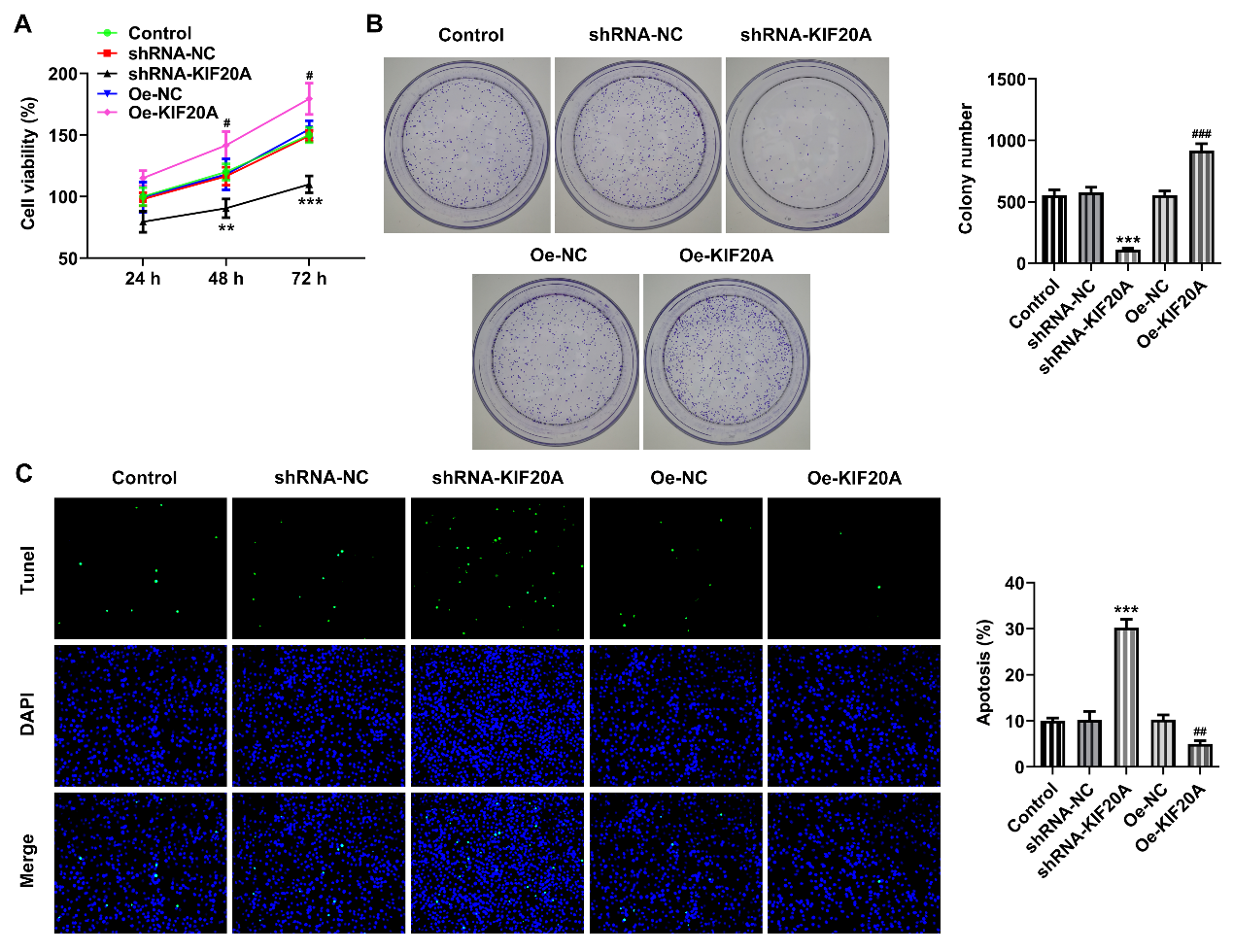
**

**FigS3:** Interference with KIF20A inhibits proliferation of ccRCC cells. A. CCK-8 detected the cell viability. B. Clone formation assay detected the cell reproductive capacity. C. TUNEL assay detected the apoptosis of cells. *P<0.05, ***P<0.001 vs shRNA-NC. # P<0.05, ## P<0.01, ### P<0.001 vs Oe-NC.

**
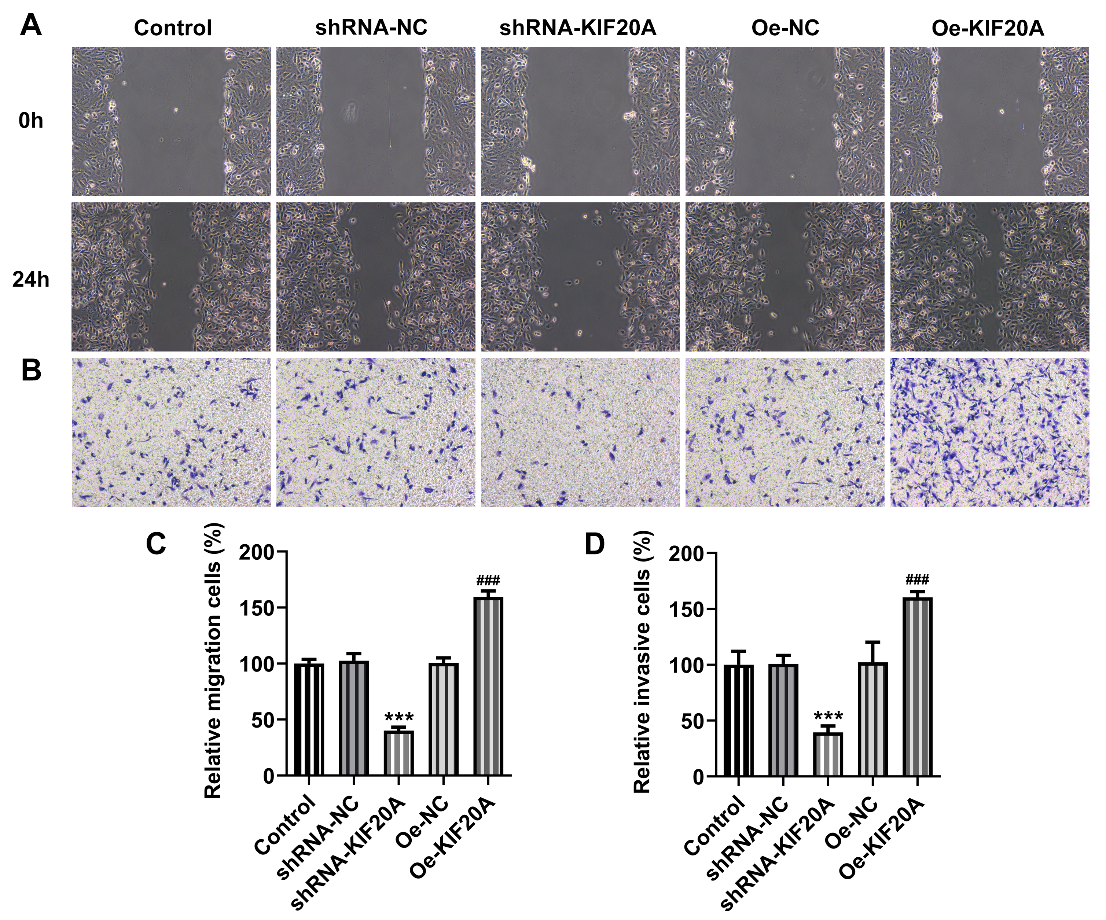
**

**FigS4:** Overexpression of KIF20A inhibits invasion and migration of ccRCC cells. A. Wound healing detected the cell migration ability. B. Transwell detected the cell invasion ability. C. Statistical chart of cell mobility. D. Statistical chart of cell invasion. ***P<0.001 vs shRNA-NC. ### P<0.001 vs Oe-NC.
