# Supplementary material for: Inhibition of KIF20A by transcription factor IRF6 affects the progression of renal clear cell carcinoma
Source: Cancer Cell Int. 2021 May 3;21:246. doi: 10.1186/s12935-021-01879-y (PMC8091794; doi:10.1186/s12935-021-01879-y)
Supplement: Supplementary file 2 — Additional file 2. Original dates. [file 12935_2021_1879_MOESM2_ESM.pptx]

## Slide 1
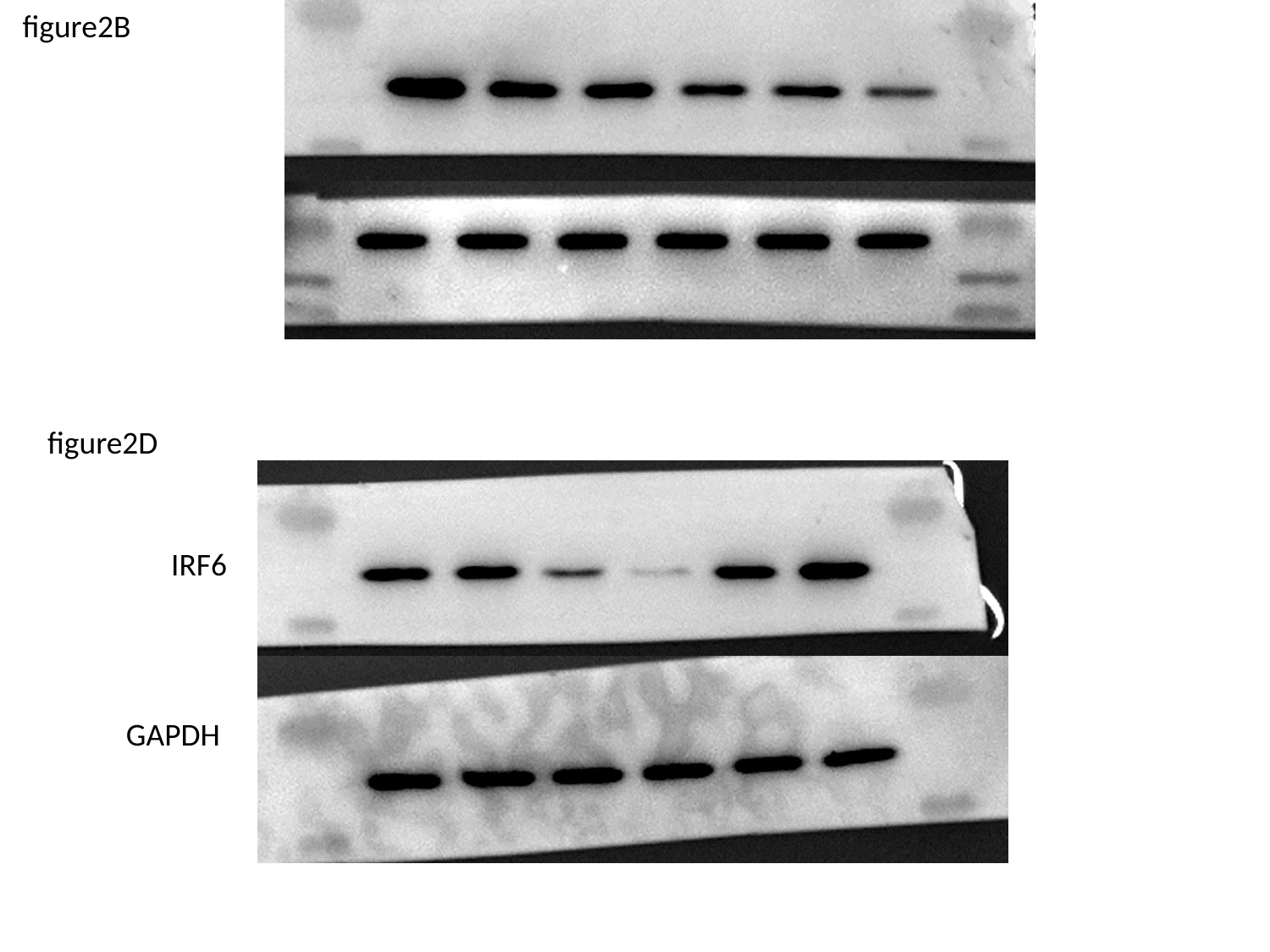

figure2B
figure2D
IRF6
GAPDH

## Slide 2
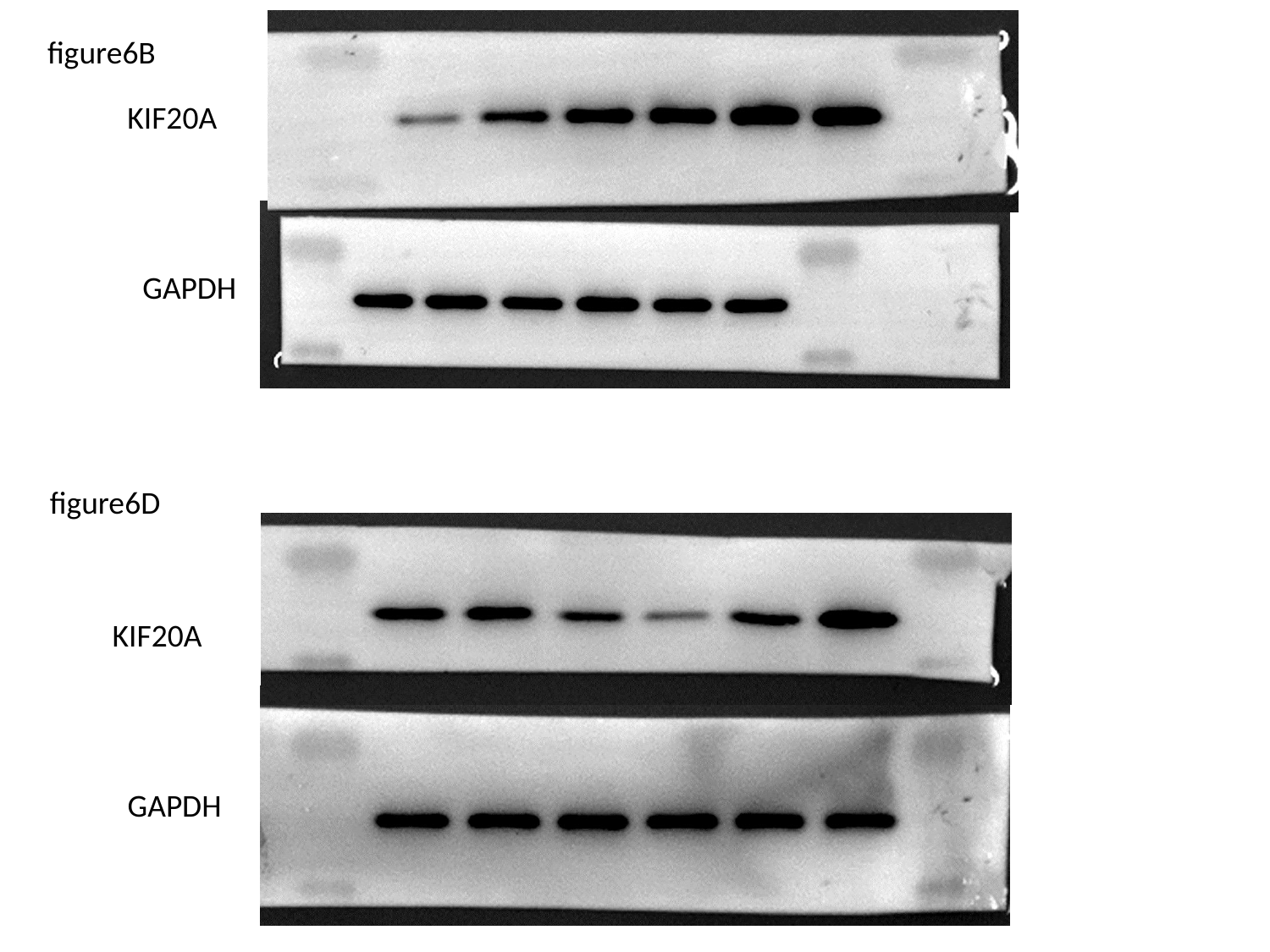

figure6B
KIF20A
GAPDH
figure6D
KIF20A
GAPDH

## Slide 3
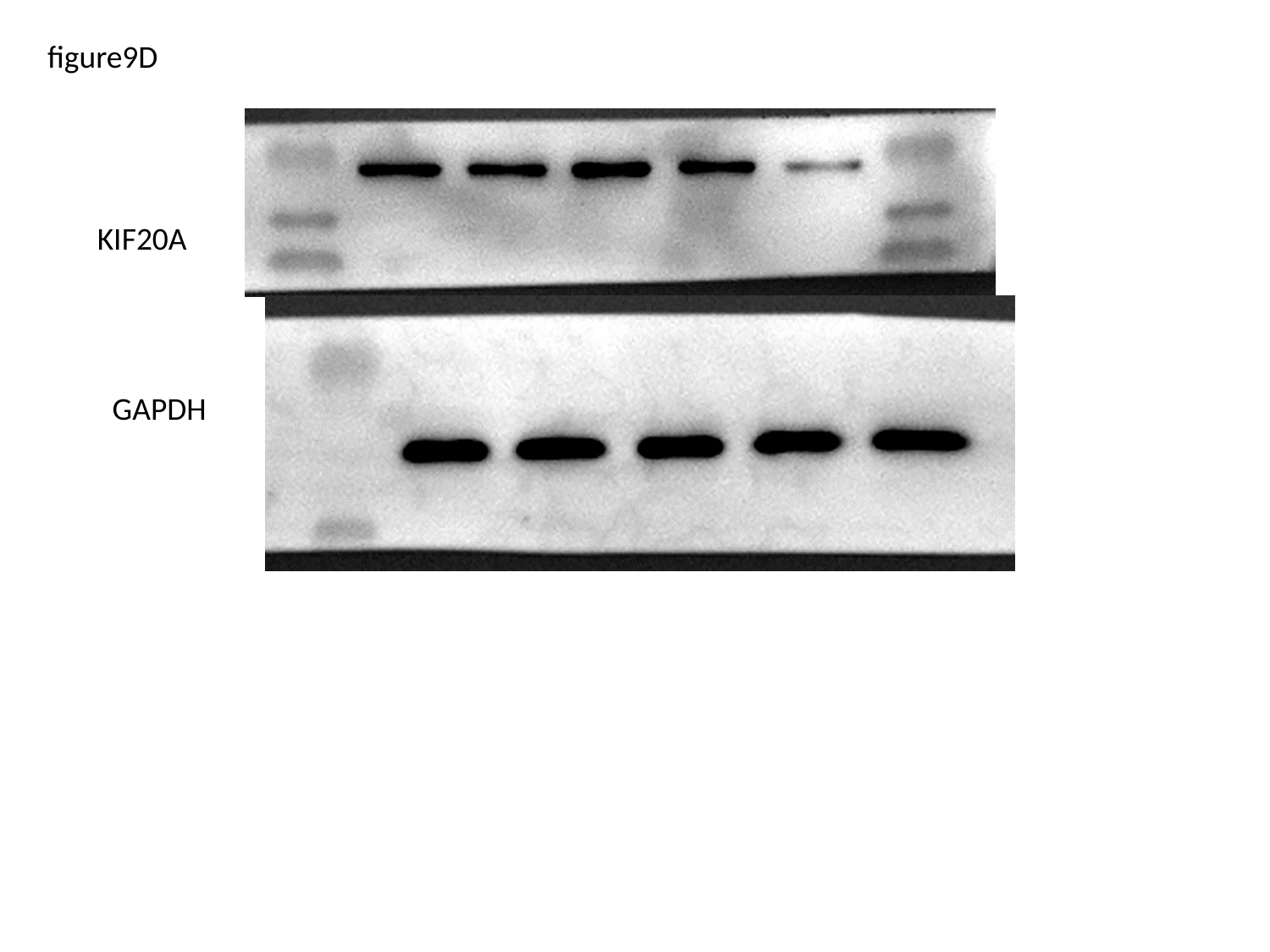

figure9D
KIF20A
GAPDH

## Slide 4
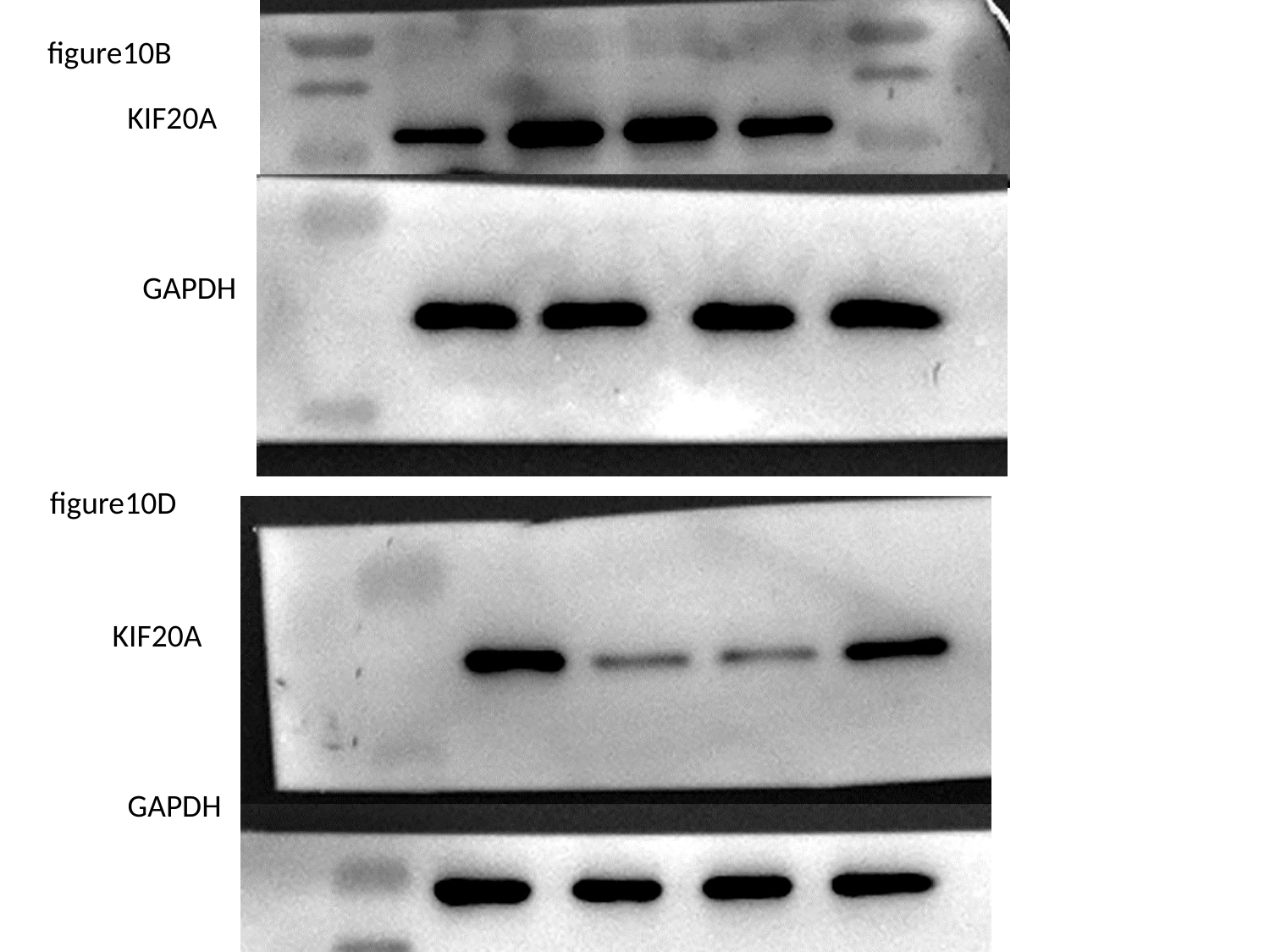

figure10B
KIF20A
GAPDH
figure10D
KIF20A
GAPDH

## Slide 5
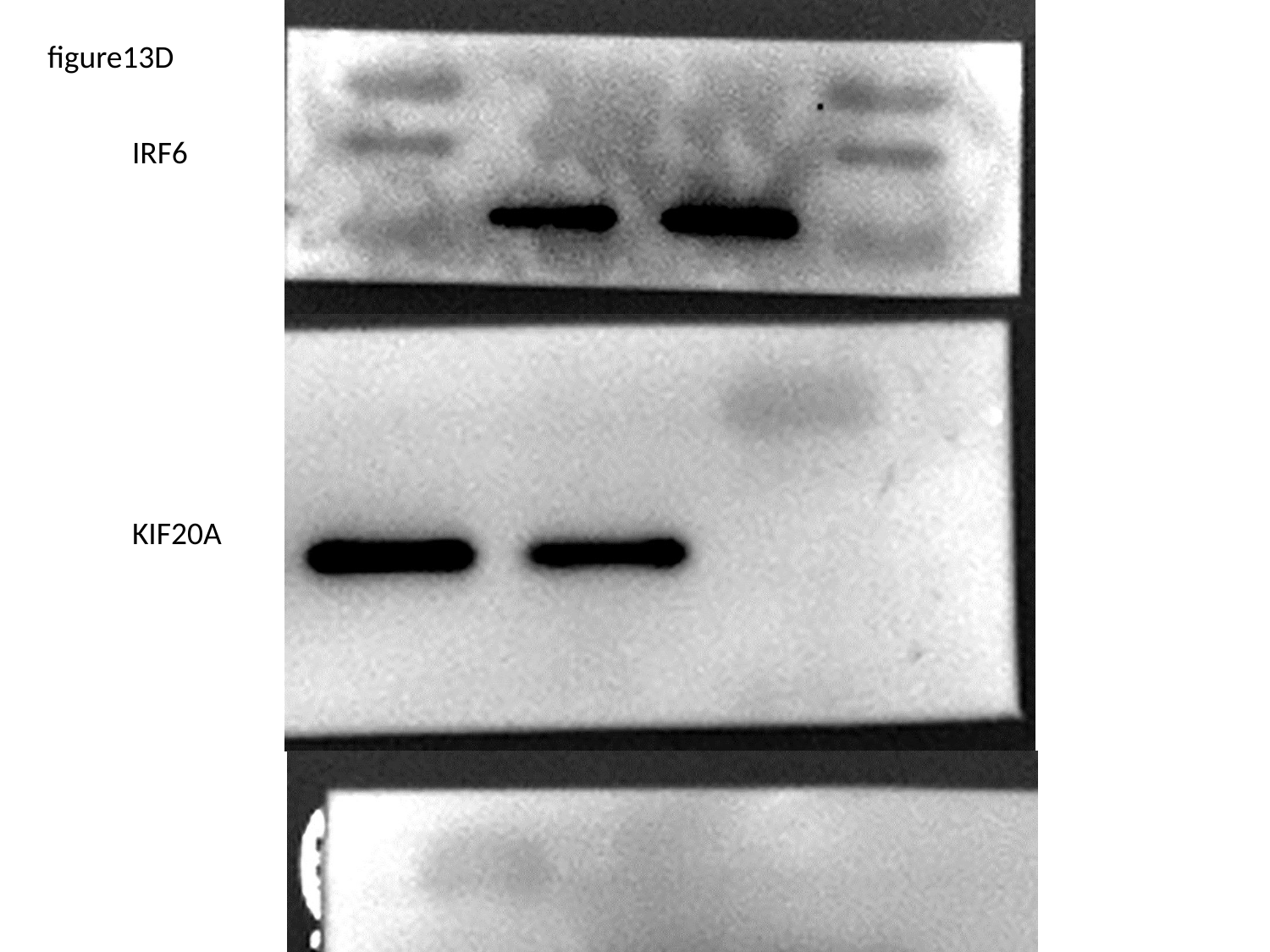

figure13D
IRF6
KIF20A
GAPDH

## Slide 6
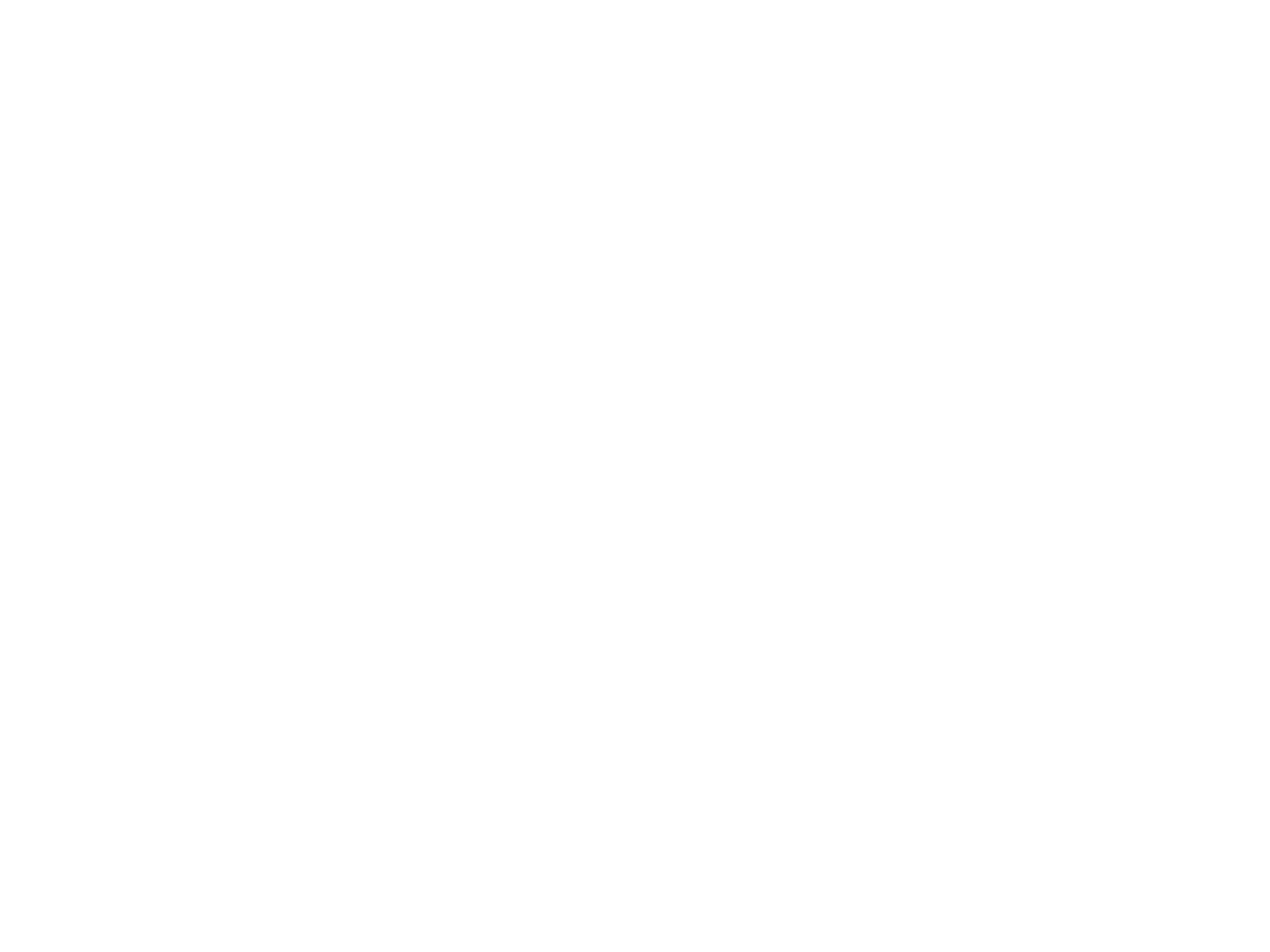

#
